# Usefulness of real time PCR to quantify parasite load in serum samples from chronic Chagas disease patients

**DOI:** 10.1186/s13071-015-0770-0

**Published:** 2015-03-12

**Authors:** Myllena F Melo, Otacilio C Moreira, Priscila Tenório, Virginia Lorena, Izaura Lorena-Rezende, Wilson Oliveira Júnior, Yara Gomes, Constança Britto

**Affiliations:** Laboratório de Biologia Molecular e Doenças Endêmicas, IOC /Fiocruz, Av. Brasil, 4365. Pavilhão Leônidas Deane, sala 209. Manguinhos, Rio de Janeiro, Brazil; Departamento de Imunologia, Centro de Pesquisas Aggeu Magalhães-CPqAM /Fiocruz, Recife, PE Brazil; Ambulatório de doença de Chagas e Insuficiência Cardíaca do Pronto Socorro Cardiológico de Pernambuco (PROCAPE), Universidade de Pernambuco (UPE), Recife, PE Brazil; Programa Integrado de Doença de Chagas/Fiocruz, Rio de Janeiro, RJ Brazil

**Keywords:** Molecular diagnosis, Real Time PCR, Chagas disease, Serum samples

## Abstract

**Background:**

Inconclusive results of serological diagnosis in Chagas disease have an important impact on blood banks worldwide, reflecting in the high number of discarded bags or in an increased transmission by blood transfusion. Molecular techniques such as qPCR have been used for diagnosis and to monitor *Trypanosoma cruzi* load in peripheral blood samples. A promising perspective refers to the possibility of parasite DNA detection in serum, taking advantage in using the same samples collected for serological screening.

**Methods:**

In order to evaluate the effectiveness of a qPCR strategy for detecting and quantifying *T. cruzi* DNA in serum, we selected 40 chronic Chagas disease patients presenting different clinical manifestations: Cardiac (23), Digestive (4), Mixed form [cardiodigestive] (7), and asymptomatic (6). Twenty seronegative individuals from non-endemic areas were included as controls. Samples were extracted using QIAamp DNA mini kit (QIAGEN) and qPCR was performed in a multiplex format with TaqMan probes for the nuclear satellite DNA of *T. cruzi* and for the human RNase P gene. In addition, DNA migration to serum during blood coagulation was assessed using a commercial exogenous control (Exo IPC, Applied Biosystems) in a separate qPCR reaction.

**Results:**

The comparative duplex qPCR analysis revealed that, even with an increase in Ct values, it was possible to detect all DNA targets in serum. In addition, the same linearity range for *T. cruzi* quantification (from 10^5^ to 0.5 par. eq./mL) between serum, blood or culture samples (*T. cruzi* epimastigotes – Cl Brener strain) was found. When patient samples were evaluated, no significant differences in parasite load between the distinct clinical manifestations were found for both blood and serum samples. Moreover, median values of parasite burden were 1.125 and 1.230 par. eq./mL for serum and blood, respectively. Using serology as gold standard, we found 95% sensitivity for *T. cruzi* detection in serum and 97.5% for blood, and 100% specificity for both samples.

**Conclusions:**

Taken together, our data indicate the potential of using serum samples for molecular diagnosis and parasite load quantification by qPCR, suggesting its use in reference laboratories for the diagnosis of Chagas disease patients.

## Background

Chagas disease is caused by the protozoan *Trypanosoma cruzi* and represents the third-highest parasitic disease burden after malaria and schistosomiasis [1,2]. It remains a relevant and endemic public health issue in 21 endemic countries of America, with an estimated 8 million people already infected and about 50,000 new cases per year [3,4]. In addition, cases of Chagas disease due to blood transfusion and organ transplantation have been increasingly detected in the United States, Canada, many European countries and Oceania, as consequence of intense migration of Latin America infected individuals to non-endemic countries [5,6]_*.*_ In this context, there is an urgent need for improving the surveillance on neglected tropical diseases for reducing the elevated number of undiagnosed Chagas disease cases in these countries [7,8].

The current diagnostic methods for Chagas disease are not satisfactory [3], being performed through the detection of parasites (or parts of its content) in blood or the presence of parasite-specific antibodies in patient’s serum [9]. The results obtained by serological tests are often inconclusive and available kits are expensive and present rather low reproducibility, due to the use of non-defined reagents. In general, serological doubtful results can bring a significant impact in blood bank screening leading to an increment of discarded blood bags. In Brazil, it is estimated 55,000 seropositive donors [10,11] and the risk of transfusion transmission via contaminated blood is about 12% - 25% [12]. In the last decade, concerning the blood banks of Pernambuco state, the proportion of inconclusive serology by two or more tests (indirect haemagglutination, indirect immunofluorescence and enzyme-linked immunosorbent assays) was higher than the concordance of positive results obtained by these tests [13]. In this sense, more reliable diagnostic methods for Chagas disease as well as biomarkers for assessment treatment response are urgently needed [9,14]. Quantitative PCR (qPCR) based assays could fill these gaps providing higher sensitivity and specificity than conventional methods to detect and quantify *T. cruzi* DNA in Chagas disease patients [15-18].

Herein, we evaluated the effectiveness of a duplex qPCR strategy based on TaqMan probes for detection and quantification of *T. cruzi* load in serum samples from chronic Chagas disease patients. Comparing the results obtained with peripheral blood samples, the data indicated the potential of using serum samples for molecular diagnosis and parasite load quantification, thus suggesting the use of qPCR in reference laboratories for the diagnosis of Chagas disease patients.

## Methods

### Ethics, consent and permissions

Forty seropositive Chagas disease chronic patients (aged 18 to 73 with a median of 62 years; 22 females and 18 males) assisted at the Ambulatório de doença de Chagas e Insuficiência Cardíaca do Pronto Socorro Cardiológico de Pernambuco (PROCAPE) – Universidade de Pernambuco (UPE), Brazil participated in the study. The serodiagnosis to confirm the infection was performed by both, conventional and recombinant enzyme-linked immunosorbent assays–CHAGAS TEST ELISA III (ABBOT/BiosChile - Bioschile Ingenieria Genética S.A., Santiago, Chile) and Imuno-Elisa CHAGAS (WAMA Diagnóstica, São Carlos, São Paulo, Brasil), following the recommendation of the Ministry of Health of Brazil (Technical Note 03/06-CGLAB/CGDT/DEVEP/SVS/MS) [9,19]. Patients were classified accordingly to their clinical manifestations as follows: cardiac (twenty three); digestive (four); cardiodigestive (seven); asymptomatic (six) [9]. Additionally, 20 individuals without *T. cruzi* infection (negative serology) living in non-endemic Chagas disease areas were included as control group. The individuals recruited for this study did not receive any blood transfusion or organ transplantation prior to blood harvesting. The study was approved by the ethical committee of the Centro de Pesquisas Aggeu Magalhães from Fiocruz (CAEE: 0096.0.095.000-07), following the principles expressed in the Declaration of Helsinki. Chagas disease patients and healthy individuals participated as volunteers and agreed to the “Terms of Free and Informed Consent”; written informed consents were obtained.

### DNA extraction from human blood and serum

For each individual, ten milliliters of venous blood were collected. Five milliliters were immediately transferred to a BD Vacutainer® Plus Plastic Serum tube and remained resting for 30 min at room temperature, in order to generate the clot from blood cells and the serum phase. The five mL remaining blood were transferred to a BD Vacutainer® Plus Plastic EDTA K3 tube. DNA extraction was performed from 200 μL of serum and blood samples, as described by Moreira et al. [20] using the QIAamp DNA Mini kit (Qiagen, Valencia, CA). The DNA eluate was stored at - 20°C until use in qPCR analysis.

### Quantitative duplex Real-Time PCR (qPCR)

The qPCR reactions were carried out with 5 μL DNA; 2X TaqMan® Universal PCR Master Mix AmpErase® from Applied Biosystems; 300nM cruzi1 (5′ASTCGGCTGATCGTTTTCGA3′) and cruzi2 (5′AATTCCTCCAAGCAGCGGATA3′) primers and 100nM cruzi3 probe (5′FAM-CACACACTGGACACCAA-NFQ-MGB3′) specific for the satellite region of the nuclear DNA of *T. cruzi*; 0,5X TaqMan RNAseP Control Reagents kit (VIC/TAMRA) from Applied Biosystems, in a final volume of 20 μL [15]. Cycling conditions were a first step at 95°C for 5 min, followed by 40 cycles at 94°C for 15 sec and 58°C for 1 min. The amplifications were carried out in an ABI Prism 7500 Fast device (Applied Biosystems, USA). Standard calibration curves for blood and serum were constructed by serial dilution of DNA extracted from blood samples spiked with *T. cruzi* epimastigotes (CL-Brener), ranging from 10^5^ to 0.5 parasite equivalents per milliliter of blood (par. eq./mL). For serum samples, whole blood was spiked with *T. cruzi* prior to serum preparation and DNA extraction.

### Evaluation of DNA migration to serum after blood coagulation

In order to verify DNA migration to serum after blood coagulation, the TaqMan® Exogenous Internal Positive Control Reagents (Exo-IPC DNA) from Applied Biosystems were used. This kit contains a synthetic DNA, without homology with any DNA sequence available on public databanks, together with a set of specific primers and TaqMan probe (VIC/TAMRA). Five milliliters of whole blood samples were spiked with Exo-IPC DNA or *T. cruzi* CL-Brener epimastigotes to reach a final concentration of [1X] or 10 parasites/mL respectively, prior to blood coagulation and serum isolation. DNA extraction and qPCR assays were conducted as described above.

### Statistical analysis

Experiments were performed in five replicates. Data were expressed as arithmetic means or average ± standard deviation. Student’s *t*-test or Mann–Whitney Rank Sum test were used to analyze the statistical significance of the observed differences. A p-value of less than 0.05 was considered statistically significant. All the analyses were performed with Sigmaplot for Windows Version 12.0 (Systat Software, Inc.).

## Results

**Figure 1:**
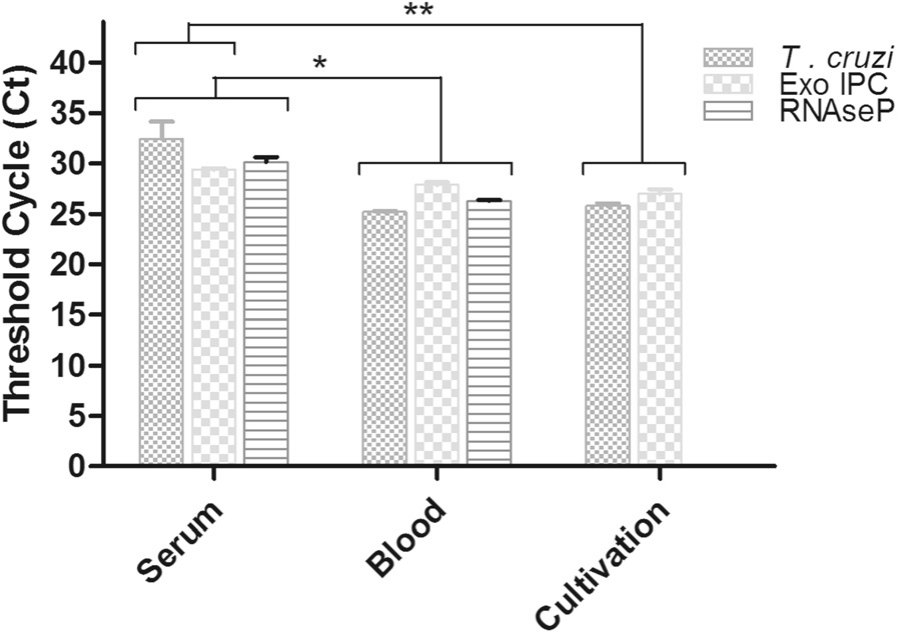
**DNA detection in serum, blood and** ***T. cruzi*** **samples by Real Time qPCR.** Whole blood was spiked with Exo-IPC (1X) or *T. cruzi* cells (1 par. eq./ mL) before serum preparation. Results are expressed as the media of Threshold cycles (Ct) and represent the average from five replicate samples ± Standard deviation. *, ** p < 0.05.

In this study the potential of serum samples to be used for the detection and quantification of *T. cruzi* load by real time qPCR was evaluated. At first, to evaluate the DNA migration to serum after blood coagulation, serum, blood and *T. cruzi* cultivated samples were compared by analyzing the resultant Ct values in multiplex qPCR assays, where the human RNase P was used as an internal amplification control [15,20] , and Exo-IPC was used as an exogenous positive control (Figure 1). For this experiment *T. cruzi* epimastigotes (CL Brener) were used to contaminate a seronegative blood sample in order to reach final concentration of 10 parasites/mL in whole blood, before serum preparation. Following blood coagulation and serum obtaining, it was observed that *T. cruzi*, Exo-IPC and RNAse P targets were detected in DNA extracted from serum samples (Cts means were 32.48, 29.38 and 30.15, respectively), showing the migration of parasite, exogenous and human DNAs to serum during blood coagulation. Nevertheless, these Ct values were higher in serum than in blood or cultivated *T. cruzi* samples (p < 0.05; Ct means for blood samples: 25.22 [*T. cruzi*]; 27.90 [Exo-IPC] and 26.31 [RNAseP] and Ct means for *T. cruzi* cultivated samples: 25.79 [*T. cruzi*] and 27.02 [Exo-IPC]. Even with an increase in Ct values, these results indicated the possibility of parasite detection in serum samples by qPCR.

**Figure 2:**
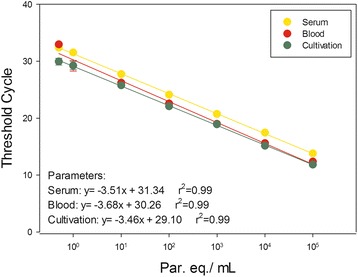
**Reportable range for** ***T. cruzi*** **detection and quantification by Real Time qPCR.** TaqMan qPCR was carried out with serial diluted DNA extracted from serum, blood or parasite cultivated samples, ranging from 10^5^ to 0.5 par. eq./mL, tested in five replicates each. The equations obtained by linear regression are indicated in the chart.

Aiming to compare the reportable range of *T. cruzi* DNA amplification between blood and serum samples, three independent standard curves were constructed ranging from 10^5^ to 0.5 par. eq./mL with DNAs extracted from spiked blood; serum derived from artificially contaminated blood; and *T. cruzi* DNA from cultured CL Brener epimastigotes (Figure 2). Through linear regression analysis, an increased linearity was observed (R^2^ = 0.99) and adequate slopes (−3.51, −3.68 and −3.46 for serum, blood and *T. cruzi* cultivated samples, respectively) for parasite quantification. The standard curve from serum samples showed higher Ct values than the ones yielded for blood and cultivated *T. cruzi* samples, thus revealing lower parasite DNA concentration in serum. Nevertheless, linearity and curve slopes were equal or higher respectively, than those observed for blood standard curve, indicating the potential of serum samples to be used in the quantification of *T. cruzi* DNA by Real Time qPCR.

In order to compare the sensitivity and specificity of the duplex Real Time qPCR between serum and blood, assays were carried out with a panel of clinical samples from 40 chronic Chagas disease patients and 20 seronegative control patients. Twenty two individuals from the Chagas disease group were female (mean age 57.77 years) and 18 individuals were male (mean age 51.61 years). To calculate the sensitivity and specificity values, serology was considered as gold standard. For each individual, two serological tests using distinct antigens preparation were performed (conventional and recombinant ELISA), and the results were concordant between them. The qPCR showed sensitivities of 97.5 and 95% for *T. cruzi* DNA detection in blood and serum samples, respectively, and 100% specificity for both samples (Table 1).

**Table 1:** **Sensitivity and specificity of the real time qPCR in blood and serum samples**

	**Blood samples**		**Serum samples**	
	**ELISA +**	**ELISA -**	**ELISA +**	**ELISA -**
qPCR +	39 (97.5%)	0 (0%)	38 (95%)	0 (0%)
qPCR -	1 (2.5%)	20 (100%)	2 (5%)	20 (100%)
Total	40	20	40	20

To calculate Sensitivity and Specificity values, serology was considered as gold standard.

**Figure 3:**
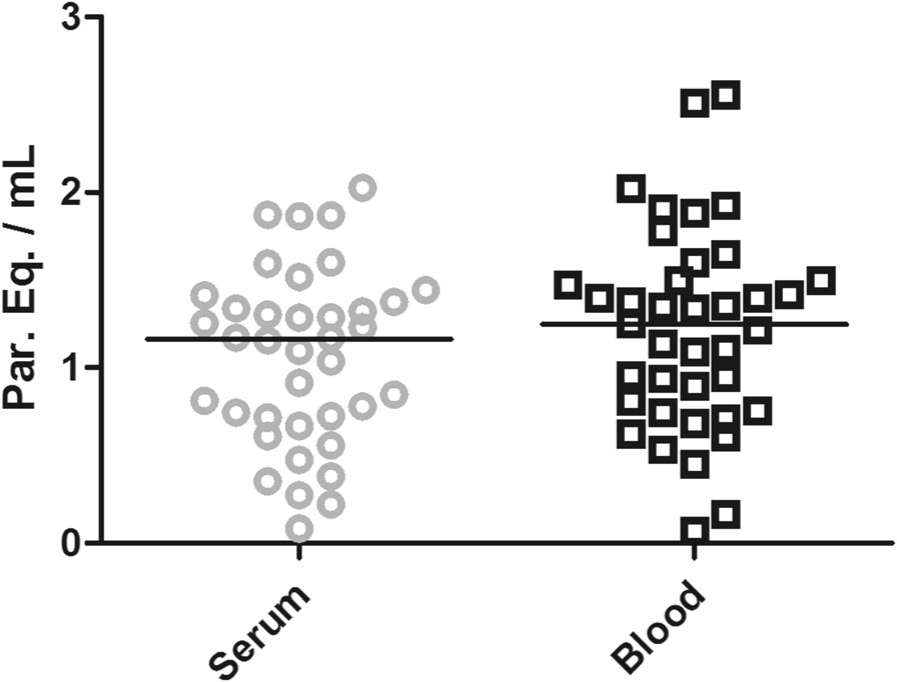
**Comparison between parasite loads in serum and blood samples from chronic Chagas disease patients.** Gray circles and black squares represent *T. cruzi* quantification in serum and blood respectively, by duplex Real Time qPCR assays. Median values are indicated by horizontal lines.

To estimate parasite load through the multiplex Real Time PCR for absolute quantification, the correspondent standard curves were used for blood and serum samples, as described in Material and Methods section. For blood samples, parasite load varied from 0.067 to 2.553 with a median of 1.23 par. eq./mL. In comparison, for serum samples, the quantification varied from 0.085 to 2.03 with a median of 1.12 par. eq./mL (Figure 3). No significant difference was observed between the median values for these samples. As a matter of fact, the estimated parasite loads for these chronic patients were very low and near the inferior limit of the qPCR dynamic range, which can interfere in the precision of *T. cruzi* quantification.

**Figure 4:**
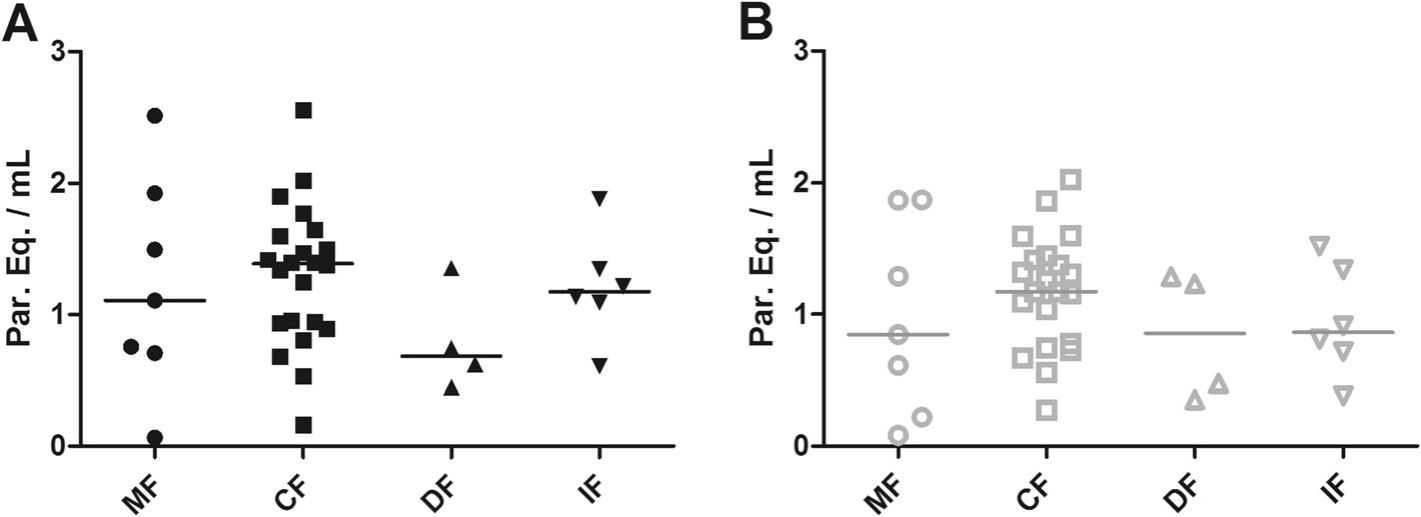
**Distribution of parasite loads in blood and serum between chronic patients with distinct clinical manifestations of Chagas disease. A**. Black symbols represent parasite loads quantified in blood samples by multiplex Real Time qPCR. **B**. Gray symbols represent the respective parasite loads in serum samples. Median values are indicated by a straight horizontal line. MF = Mixed Form (n = 7); CF = Cardiac Form (n = 23); DF = Digestive Form (n = 4); IF = Indeterminate Form (n = 6).

Patients were divided into 4 groups, according to the clinical manifestation of Chagas disease: Cardiac Form (CF = 23), Digestive Form (DF = 4), Indeterminate Form (IF = 6) and Mixed Form (MF = 7). When patients were analyzed according to their clinical manifestations, no significant difference was observed in the parasite load medians between the distinct forms of the disease, even in blood (MF = 1.11, CF = 1.39, DF = 0.68, IF = 1.18 par. eq./mL) or serum (MF = 0.85, CF = 1.17, DF = 0.85, IF = 0.86 par. eq./mL) (Figure 4). Nevertheless, some patients presenting mixed (cardiodigestive) and cardiac forms showed a slight increase in parasitemia in both types of samples. However, as observed in Figure 3, all parasite loads were very low and can compromise the quantification precision.

Although the number of patients evaluated in this study is quite small, our data raised a discussion regarding the application of Real Time qPCR in serum samples for the diagnosis and quantification of *T. cruzi*, in order to follow-up Chagas disease patients, especially in situations where serum is the only available sample.

## Discussion

Currently, WHO recommends the use of 2 simultaneous serological tests for the diagnosis of chronic Chagas disease, as indirect haemagglutination, indirect immunofluorescence and enzyme-linked immunosorbent assays, based on detection of parasite-specific antibodies [9]. However, there is an elevated number of inconclusive results in blood banks worldwide, resulting in blood bag losses and increasing the risk of transmission by blood transfusion [5,6,8]. The Brazilian guideline for chronic Chagas disease diagnosis determines the use of PCR to confirm inconclusive serological tests, resulting in a new blood sample collection [9]. In this context, we aimed to investigate the feasibility of Chagas disease molecular diagnosis and parasite load quantification using the same serum sample yielded for serological tests, based on previous studies that confirmed the use of serum samples for *T. cruzi* DNA detection by PCR [21]. Nevertheless, from our knowledge, this is the first report for using a multiplex Real Time qPCR assay able to detect and quantify *T. cruzi* from serum samples.

In this study, it is evident that the migration of *T. cruzi* DNA to serum occurs after blood coagulation. When we spiked whole blood with an exogenous DNA or living *T. cruzi* epimastigotes prior to serum isolation, both DNAs could be detected by multiplex Real Time PCR assay, in conjunction with human DNA from nucleated blood cells. As expected, the higher Ct values observed in serum, in comparison with blood and parasite cultivated samples, indicated that only part of parasite and human DNA migrates to serum after blood coagulation. Nevertheless, the sensitivity for *T. cruzi* detection in chronic Chagas disease patients was only slightly lower in serum (95.0%) than blood (97.5%), and the same specificity (100%) was observed for both samples (Table 1), which makes it possible to use serum samples to detect *T. cruzi* DNA by Real Time qPCR. Using serological methods as gold standard, clinical sensitivity for conventional and Real Time PCR assays presents great variability among distinct studies [17,22-26]. In addition, using cruzi1/cruzi2 primers and cruzi 3 TaqMan probe, Duffy et al. [17] reported sensitivity values ranging from 60.3 – 100% for T. cruzi detection in blood samples from patients from different geographical areas (Venezuela, Cochabamba, Argentina and North Argentina), infected by distinct transmission routes (oral infection, chronic Chagas disease and newborns from chronic Chagas disease mothers), and presenting different parasite load medians (2.75 – 691.8 parasite equivalents/ mL). On the other hand, Fitzwater et al. [26] reached 60.1%, 46.5% and 40% sensitivity from clot, buffy coat and whole blood samples, respectively, in a conventional PCR assay using 121/122 primers. Indeed, multiple factors seem to influence these results, as the primers used (kDNA vs Sat-DNA), DNA extraction method, PCR reagents, T. cruzi genotype (DTUs) and parasite load. Following our methodology, even considering low parasite loads, we reached high and similar sensitivity values for blood and serum.

Nowadays, efforts are being done to standardize Real Time qPCR for *T. cruzi* quantification in blood samples. Following the recommendations from the international workshop promoted by PAHO/WHO in 2011, Duffy *et al.* [17] reported the analytical performance of a Multiplex Real-Time qPCR for the quantification of *T. cruzi* satellite DNA in blood samples. They observed a reportable range between 1 and 6 log_10_ par. eq./10 mL for a TcI isolate (Silvio X10) and 0.25 to 6 log_10_ par. eq./10 mL for a TcVI isolate (CL Brener). In addition, for the CL Brener isolate, it was observed a Limit of Detection (LOD) and Limit of Quantification (LOQ) of 0.6979 and 1.531 par. eq./mL, respectively. Herein, using the same set of primers for *T. cruzi* nuclear satellite DNA multiplexed with human RNAse P gene instead of using the Internal Amplification Control (IAC), we observed a reportable range from 10^5^ to 0.5 par. eq./mL (Figure 2) for DNAs extracted from serum, blood or *T. cruzi* cultivated samples (CL Brener). Likewise, our results were feasible for parasite load quantification in Chagas disease patients with moderated parasitemia.

In this study, the low parasite load achieved in blood or serum samples reproduced the findings observed in recent studies regarding the use of qPCR for chronic Chagas disease patients [16,17,20]. The parasite load median in blood (1.23 par. eq./mL) was not statistically different from the one achieved from serum samples (1.12 par. eq./mL) (Figure 3), which corroborates the use of DNA extracted from serum for qPCR assays. Nevertheless, comparing with the LOQ previously determined [17], several patients herein analyzed, presented parasite load below the LOQ, which means these samples were detectable but not quantifiable. When patients presenting distinct clinical manifestations were compared (Figure 4), we also could not observe significant differences between the parasite load medians from the groups evaluated, although many patients presenting the cardiac form (CF) seems to have higher parasite load in blood and serum samples. Unfortunately, in this study, there was a limitation concerning the number of analyzed patients, particularly for those with the digestive form of the disease. In the future, a robust investigation joining a representative group of patients with all Chagas disease clinical manifestations will be necessary to properly investigate parasite load as a biomarker for progression and severity of Chagas disease. The advances achieved here suggest the use of serum samples, currently maintained in serum banks, for retrospective studies regarding the disease development and to the follow-up of patients under treatment. Meanwhile, a new study with considerable higher numbers of Chagas disease patients, especially those asymptomatic individuals who can act as potential blood donors, must be performed to validate this molecular diagnostic assay in blood banks worldwide.

## Conclusions

Taken together our data suggest the potential of serum samples to be used for molecular diagnosis and parasite load quantification by quantitative Real Time PCR assays.
